# Review of Requirements for the Durability and Damage Tolerance Certification of Additively Manufactured Aircraft Structural Parts and AM Repairs

**DOI:** 10.3390/ma13061341

**Published:** 2020-03-15

**Authors:** Sudip Kundu, Rhys Jones, Daren Peng, Neil Matthews, Alankar Alankar, Singh R. K. Raman, Pu Huang

**Affiliations:** 1 Centre of Expertise for Structural Mechanics, Department of Mechanical and Aerospace Engineering, Monash University, Clayton, Victoria 3800, Australia; sudip.kundu@monash.edu (S.K.); daren.peng@monash.edu (D.P.); Raman.singh@monash.edu (S.R.K.R.); pu.huang@monash.edu (P.H.); 2 Department of Mechanical Engineering, Indian Institute of Technology Bombay, Powai, Mumbai, Maharashtra 400076, India; alankar.alankar@iitb.ac.in; 3 RUAG Australia, 836 Mountain Highway, Bayswater VIC 315, Australia; Neil.matthews@ruag.com; 4 Department of Chemical Engineering, Monash University, Clayton, Victoria 3800, Australia

**Keywords:** additive manufacturing, friction stir welding, laser shock peening, cold spray, residual stress, multiple collocated cracks

## Abstract

The USAF requirements for the durability and damage tolerance certification for additively manufactured (AM) aircraft structural parts, which are detailed in Structures Bulletin EZ-19-01, raise a number of new and, as yet, unanswered questions. The present paper attempts to address three questions: How to perform a fracture mechanics-based analysis of crack growth in an AM part so as to account for the residual stresses, how to perform a fracture mechanics-based durability analysis of a cold spray repair so as to account for both the induced residual stresses and the presence of multiple co-located cracks, and how to perform a fracture mechanics-based durability analysis of an AM part so as to account for the presence of multiple collocated surface braking cracks. In this context, the present paper reveals the potential of the Hartman–Schijve variant of the NASGRO crack growth equation to accurately predict the growth of each of the individual (collocated) cracks that arose in a cold spray-repaired specimen and in a specimen from a crack that nucleated and grew from a rough surface.

## 1. Introduction

The March 2019 memo by the Under Secretary, Acquisition and Sustainment [1] enunciated that, as of March 21st 2019, the US Department of Defense (DoD) will use additive manufacturing (AM) to “enable the transformation of maintenance operations and supply chains, increase logistics resiliency, and improve self-sustainment and readiness for DoD forces.” This memo further stated that: “AM parts or AM repair processes can be used in both critical and non-critical applications. For all applications, the appropriate level of qualification, certification, and risk/safety evaluation must be completed by the appropriate engineering support activity.” This statement represents a significant development since, until recently, the focus has been on “low hanging fruit,” i.e., non-critical parts that do not affect the safety of flight. Indeed, as implied in [1] and as reflected in [2,3], AM offers the potential for the “on-demand” manufacturing of structural parts, albeit with a life that may be less than the original design life but nevertheless sufficient to ensure continued operational capability until a conventionally manufactured replacement part can be obtained. USAF Structures Bulletin EZ-19-01 [3,4] subsequently outlined the USAF requirements for the durability and damage tolerance certification for AM aircraft structural parts and AM repairs, such as cold spray. Structures Bulletin EZ-19-01 states that for structural parts that are manufactured from an AM process, the most difficult challenge is to establish an “accurate prediction of structural performance” specific to DADT (durability and damage tolerance). Several challenges to which there are no current answers arise directly from Structures Bulletin EZ-19-10:

(i) How can we accurately compute crack growth in an AM part when there is a residual stress field?
(ii) How can we accurately compute crack growth in a cold spray repair where cracking can nucleate, either in the substructure being repaired or at the intersection between the cold spray?
(iii) How can we accurately perform a durability analysis for a cold spray repair if there are collocated cracks?
(iv) Can we relate da/dN versus ΔK equations, where da/dN is the crack growth rate and ΔK is the difference between the maximum and minimum values of the stress intensity factor in a load cycle, the crack growth rate needed to perform items (i) through (iii) to the corresponding curves determined for the conventionally manufactured material?

In this context, it should be noted that Section 5 of MIL-STD-1530D [5] explains that analysis is the key to both the damage tolerant design and the through life sustainment of USAF aircraft, and that the primary role of full scale testing is “to validate or correct analysis methods and results and to demonstrate that requirements are achieved”.

To answer these questions, a “building block” approach is adopted. It is first shown that the Hartman–Schijve variant of the NASGRO crack growth equation [6,7,8,9,10] can be used to compute crack growth in AM, and to compute the effect of residual stresses on crack growth in problems involving laser shot peening (LSP) and friction stir welding (FSW). Laser shock peening, which uses short-time (nanosecond) laser pulses to vaporize the material on the surface of a specimen, is now being widely used in the aerospace industry, and applications to both F-22 and F-35 aircraft were discussed in [11,12]. The ability of LSP to improve the fatigue life of a component is believed to be due to the residual stresses that arise from the LSP process [12,13,14,15]. As a result, LSP has been shown to be particularly effective as a post processing tool to improve the fatigue performance of additively manufactured parts [14,15]. Friction stir welding, which was invented by Wayne Thomas and colleagues at The Welding Institute in Cambridge [16,17], is also recognized [18] as a “key technology” for primary aerospace structures. Crack growth in FSW components also involves the interaction between the applied loads and the residual stresses [18,19,20].

In this context, it should be noted that it has long been known that the residual stresses resulting from the manufacturing processes can significantly influence crack growth in laser shock peened parts, AM parts, and FSW parts [18,19,20,21,22,23]. It is now known [24,25,26,27] that, for welded structures, provided that allowance is made for the effect that the residual stress intensity factor (*K*_res_) has upon the true range of the stress intensity factor (∆K_true_) and on the true value of the maximum stress intensity factor (*K*_true,max_), then crack growth can often be reasonably accurately computed. Unfortunately, there are few studies regarding whether this observation has been shown to be true for AM parts.

In this paper, we have chosen to focus on the ability of the Hartman–Schijve variant of the NASGRO crack growth equation to answer questions (i) through (iv). The reason for this is that it is now known [6,7,8,9,10,28,29,30,31,32,33,34,35,36,37,38,39,40,41,42,43,44,45,46] that the Hartman–Schijve equation can be used to study crack growth in traditionally manufactured aerospace aluminum and titanium alloys, aerospace steels, rail steels, bridge steels, adhesively-bonded metallic structures, and adhesively-bonded wood structures. The Hartman–Schijve equation has also been shown to be able to represent crack growth in cold spray repairs [46,47], AM materials [3,48,49,50], and delamination growth in polymer composites [51,52,53,54,55,56,57,58,59]. Aerospace-related examples that illustrate the ability of the Hartman–Schijve equation to accurately compute the crack growth histories that are obtained in conventionally manufactured metallic structures tested under an operational flight load spectra include:

(i) The growth of small, naturally occurring cracks in the 1969 F-111 D6ac wing test [6,32].
(ii) The growth of small, naturally occurring cracks in an aluminum alloy AA7050-T7451 plate simulating a combat aircraft wing root shear restraint (or shear tie post) subject to a combined aerodynamic buffet and maneuver load spectrum [33].
(iii) Cracking in an aluminum alloy AA7050-T7451 plate that was tested under a civil aircraft flight load spectrum (MiniTWIST) [34].
(iv) The growth of both small and long cracks in AA7075-T6 under measured maritime patrol aircraft (P3C Orion) flight load spectra [29,31].
(v) The growth of small, naturally occurring cracks in an aluminum alloy AA7050-T7451 plate that was tested under a F/A-18 flight load spectrum [6,9,33].

Furthermore, not only is it known that the Hartman–Schijve equation is able to represent crack growth in a range of AM materials, it has also been shown [3,48,49,50] that the Hartman–Schijve crack growth equation holds for AM Ti-6Al-4V regardless of whether the AM process is electron beam melting (EBM), direct metal laser sintering (DMLS), selective laser melt (SLM), hot isostatic pressing (HIP), and laser engineered net shaping (LENS). In the case of LENS Ti-6Al-4V, this has been shown to be true regardless of whether the LENS process is low- or high-power or whether the build direction is horizontal or vertical. Indeed, it has been shown [50] that the growth of small sub mm cracks in LENS Ti-6Al-4V is captured by the same Hartman–Schijve equation with the fatigue threshold set to a small value. (This finding is particularly relevant to the durability/economic life assessment of an AM replacement part where the equivalent initial size (EIDS) required is small (e.g., sub mm) [4].).

Similarly, it has been shown [46] that for cold spray repair, which is sometimes referred to as supersonic particle deposition (SPD) [47] which is also being used to repair damaged aircraft structures [46,60,61,62,63,64], the Hartman–Shijve equation can be used to compute the fastest growing crack in the substrate.

As a result of this study, we find that:

(a) The Hartman–Schijve crack growth equation has the potential to perform a fracture mechanics-based analysis of crack growth in AM materials with a known residual stress field.
(b) It can also be used to perform a fracture mechanics-based durability analysis of crack growth in cold spray repairs when there are multiple collocated cracks.
(c) It has the potential to analyze crack growth that is associated with multiple surface breaking cracks.

These findings are significant given the central role that analysis has in the certification of AM and AM repairs and that, as noted, the above role of testing is to validate or correct analysis methods.

## 2. Materials and Methods

The studies analyzed in this paper were either taken from peer reviewed journals that are publicly available or from texts that are publicly available—their ISBN number is quoted. Similarly, the references used in this paper were taken from peer reviewed journals that are publicly available, refereed conferences, texts that are publicly available (in such cases, the ISBN number is quoted), or from Google searches. Of these references, seventy seven are in journals listed in SCOPUS and World of Science (WOS), the seven books/book chapters referenced are all listed in SCOPUS, one report is on the North American Space Administration (NASA) Technical Report Server, two references are contained in the proceedings of 13th International Conference on Materials (ICM13) with the ISBN number is given, two references are available on the US Department of Defense DTIC website, one is on the NATO-RTO web site, one is on the US Federal Aviation Administration (FAA) website, and another is on the US Navy navy.mil website. Keywords that were used in these searchers were cold spray, supersonic particle deposition (SPD), durability, damage tolerance, Hartman–Schijve, friction stir wording, laser shot peening, small cracks, additive manufacturing, AM, crack growth in operational aircraft, full scale fatigue tests, and aircraft certification, among others. The exception to this is the memo from the Under Secretary, Acquisition and Sustainment [1], which is unclassified and has no release restriction; this was provided by RUAG Switzerland.

The analysis presented in this paper began by using the Hartman–Schijve variant [13] of the NASA NASGRO crack growth equation to compute the effect of the residual stress field on crack growth in an FSW Ti-6Al-4V specimen. This crack growth equation was then used in each of the subsequent analyses, viz: crack growth in a laser shot peened, AM, and cold spray-repaired specimens.

The general form of the Hartman–Schijve variant of the NASGRO equation that was used in this paper is as given in [22]:

da/dN = *D* (Δκ)*^p^* (1)

where *a* is the crack length/depth, *N* is the number of cycles, *D* is a material constant, *p* is another material constant that is often approximately 2, and *A* is the cyclic fracture toughness. The crack driving force Δ*κ* that is used in this formulation was first suggested by Schwalbe [65]:

Δκ = (Δ*K* − Δ*K*_thr_)/(1 − *K*_max_/A)^1/2^ (2)

where *K* is the stress intensity factor, *K*_max_ and *K*_min_ are the maximum and minimum values of stress intensity factor seen in the cycle, ∆*K* = (*K*_max_ − *K*_min_ ) is the range of the stress intensity factor that is seen in the cycle, and Δ*K*_thr_ is the “effective fatigue threshold.” As explained in [6,48], the terms Δ*K*_thr_ and *A* (which is commonly referred to as the cyclic fracture toughness) are best interpreted as parameters that are chosen so as to fit the measured da/dN versus ∆*K* data. As further explained in [48], the term Δ*K*_thr_ is related to the US American Society for Testing and Materials (ASTM) definition of fatigue threshold Δ*K*_th_, which is arbitrarily be taken to be the value of Δ*K*_th_ at a value of da/dN of 10^−10^ m/cycle, by:

Δ*K*_th_ = Δ*K*_thr_ + (10^−10^/*D*)^1/p^ (3)

A similar crack growth equation was presented in [66,67,68,69] and has been applied to a wide range of problems including both the growth of small surface breaking [67] and subsurface cracks [68]. The primary difference in these two related equations lies in the form of the denominator, i.e., how the increase in the crack growth rate as *K* approaches *K*_c_ (the fracture toughness) is represented; see [6] and the Appendix A for more details.

Reasons for focusing on the Hartman–Schijve equation for AM parts and additive metal repairs are:

(i) It has been shown [3,48,49,50] that the variability in crack growth due to the AM manufacturing process can be captured by allowing for the variability in the terms ΔKthr and A.
(ii) The Hartman–Schijve equation, with the threshold term ΔKthr set to a small value, is able to compute the growth of small cracks in LENS AM Ti-6Al-4V [50].
(iii) The Hartman–Schijve equation is able to compute the variability in the growth of small cracks in laser additively deposited (LAD) metal repairs [49].
(iv) It has been shown to capture the growth of the fastest cracks in cold spray repairs to cracked fastener holes in specimens that are cut from AA7075-T6 P3C Orion wing skins [46]. Here, it should be noted that its ability to capture the growth of multiple collocated cracks has not as yet been established. This is done in the present paper.

Though, as noted above, the present paper focuses on the use of the Hartman–Schijve crack growth equation, it should be noted that a range of other approaches are commonly used to study crack growth in conventionally manufactured materials [6]. For example, approaches that have been used to model crack growth in FSW include crack closure [70], AFGROW [71,72], NASGRO [20], and the two-parameter crack growth model [71]. As previously mentioned, a number of authors have suggested that fatigue crack growth in FSW joints is dominated by weld residual stress. The experimental data presented in [70,73,74] revealed that the effect of weld residual stress on crack growth is less at high R ratio than at the low R ratio, and that the da/dN versus ΔK curves associated with base metal and FSW joints tend to become closer in the Paris region. As such, it would appear that, as suggested in [9,10,18,19,20,21], the difference in the da/dN versus ΔK curves is essentially due to the residual stresses and that this difference may be accounted for by allowing for the residual stress field.

With this in mind, let us next examine whether this formulation can also capture crack growth in components with a complex residual stress field.

## 3. Crack Growth in FSW Ti-6Al-4V

**Figure 1:**
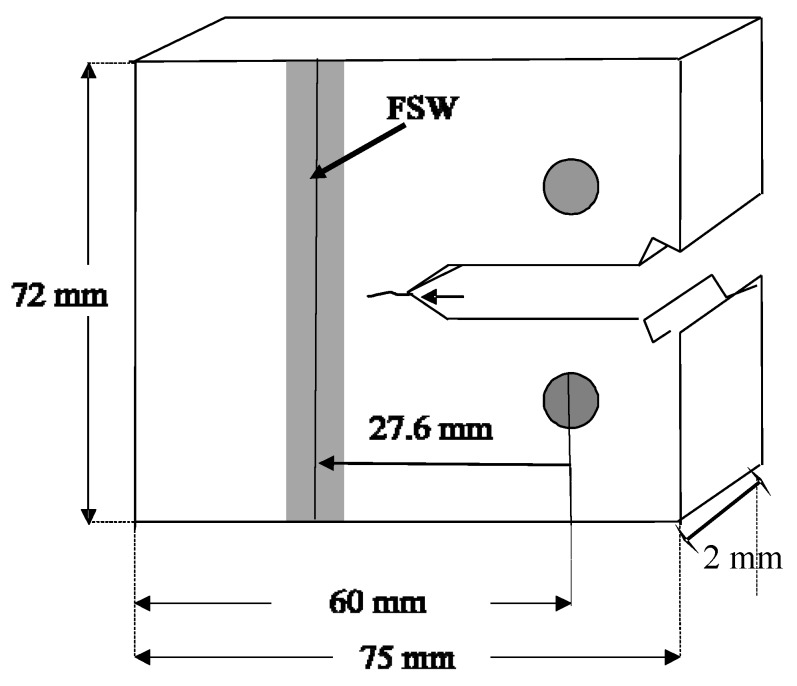
Compact tension (CT) specimen test geometry; from [72].

Let us first consider cracking in 2 mm-thick Ti-6Al-4V FSW compact tension (CT) specimens reported in [72] that were tested with an applied ΔK of 30 MPa √m. The geometry of the test specimens is shown in Figure 1, and the variation of the residual stress intensity factor *K*_res_, that resulted from FSW, is given in Figure 2 from [72].

It is now known [3,48,49,50] that the da/dN versus Δ*K* relationship for Mil-annealed Ti-6Al-4V can be expressed as:

da/dN = 2.79 × 10^−10^ [(Δ*K* − Δ*K*_thr_)/(1 − *K*_max_/*A*)^1/2^]^2.12^ (4)

**Figure 3:**
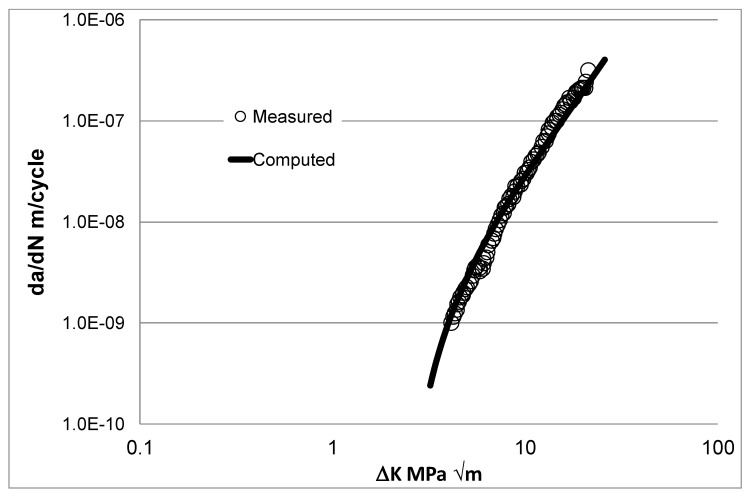
Comparison between the measured [72] and computed da/dN versus ∆*K* curve for the baseline material.

The value of the cyclic fracture toughness *A* = 87.9 MPa √m for this material was given in [72]. To estimate the value of Δ*K*_thr_ associated with *R* = 0.1 tests, we compared predictions that were made by using Equation (4) with the experimental *R* = 0.1 da/dN versus Δ*K* curve given in [72] for Mil-annealed Ti-6Al-4V. This yielded a value of ∆*K*_thr_ = 2.4 MPa √m. Figure 3 reveals a reasonably good agreement between the measured and the resultant computed da/dN versus Δ*K* crack growth curves.

Equation (4), with the values of A (=87.9 MPa √m) and ∆K_thr_ (=2.4 MPa √m) given above, was then used in conjunction with the variation in the residual stress intensity factor field as a function crack length given in [72] to compute the variation in the crack growth rate for an FSW specimen that was tested with ∆K = 30 MPa √m and R= 0.1. This analysis used the true range of the stress intensity factor, which we defined as ∆K_true_, where:

∆*K*_true_ = *K*_true,max_ − *K*_true,min_ (5)

*K*_true,max_ = *K*_max_ − *K*_res_ (6)

*K*_true,min_ = *K*_min_ − *K*_res_ (7)

and

∆*K*_true_ = *K_t_*_rue,max_ (8)

if      Δ*K*_true_ < 0 (9)

This in turn results in an effective R ratio, which we define as R_true_:

*R*_true_ = K_true,min_/K_true,max_ (10)

If the relationship between R and ΔK_thr_ is not known, then an approximate value of the threshold term ΔK_true,thr_, which corresponds to R_true_ and can be estimated by using the formulae given by McEvily and Groeger [69]:

Δ*K*_true,thr_ = Δ*K*_thr_ (*R* = 0) √((1 − *R*_true_)/(1 + *R*_true_)) (11)

where ΔK_thr_ (R = 0) is the value of the threshold term ΔK_thr_ corresponding to an R ratio of zero; see the Appendix A.

**Figure 4:**
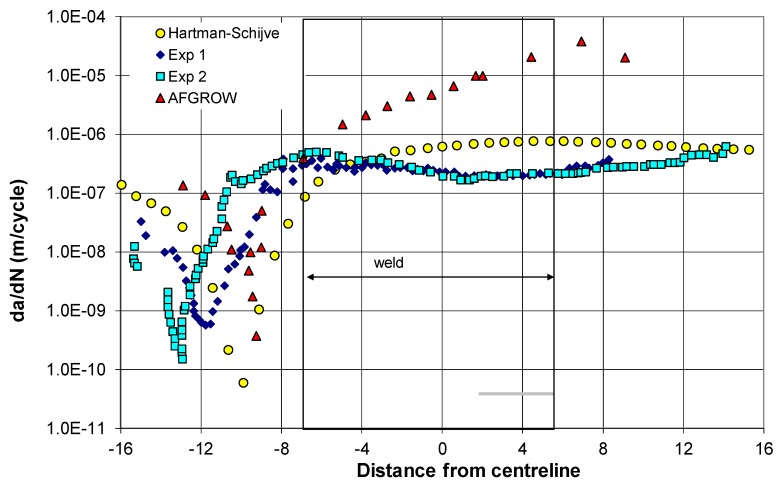
Comparison of the measured values, from [72], and computed crack growth rates.

The resultant measured [72] and predicted crack growth rate curves, obtained by using the methodology and the values outlined above, are shown in Figure 4 together with the crack growth rates that were computed in [72] by using AFGROW. In the present analysis, the residual stress field was first used to compute the value of R_true_, and Equation (11) was then used to determine the value of ΔK_true,thr_ to be used in Equation (4); see the Appendix A. Figure 4 reveals reasonably good agreement between the measured and the computed crack growth curves.

## 4. Crack Growth in Laser Shock Peened 2024-T3

**Figure 5:**
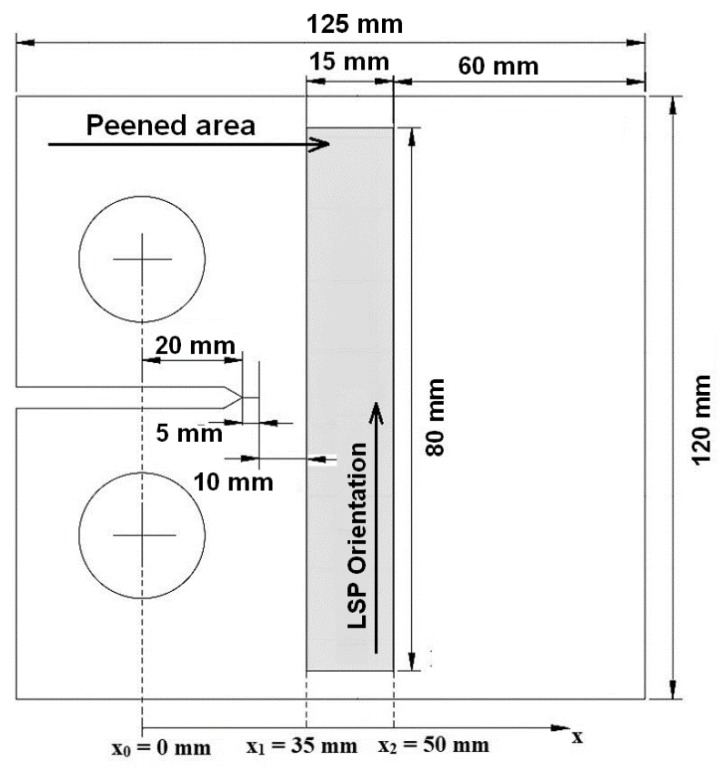
Geometry of the CT test specimen and the location of the laser shot peened (LSP) region; from [75].

**Figure 6:**
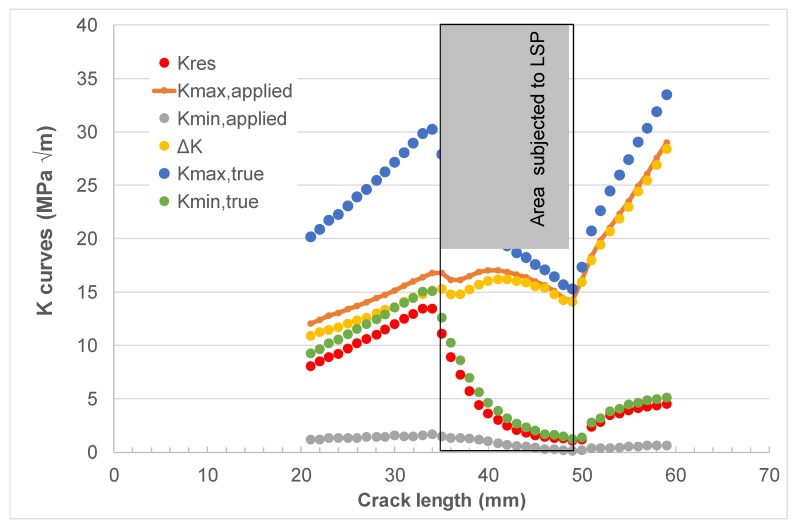
The various stress intensity factor versus crack length curves given in [75].

**Figure 7:**
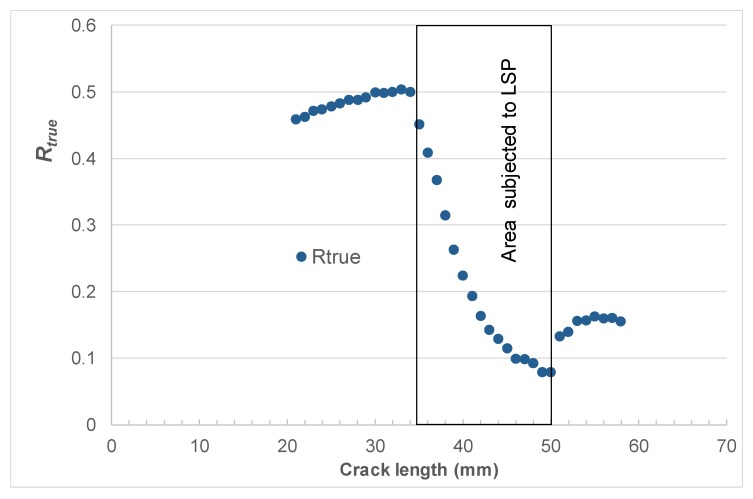
The relationship between an effective R ratio (*R*_true_) and the crack length for the LSP study reported in [75].

As previously mentioned, laser shock peening (LSP) is used on both F-22 and F-35 aircraft. The challenge is to be able to account for the residual stresses that result from the use of LSP on crack growth. To address this challenge, let us next analyze cracking in the laser shot peened 4.8 mm compact tension 2024-T3 specimens reported in [75]. The geometry of the test specimens is shown in Figure 5. The relationship between the applied stress intensity factors *K*_max_ and *K*_min_, Δ*K*, and the residual stress intensity factor *K*_res_ with crack length reported in [75] is shown in Figure 6. A plot of relationship between the true *R* ratio, *R*_true_, and crack length seen in [75] is given in Figure 7.

**Figure 8:**
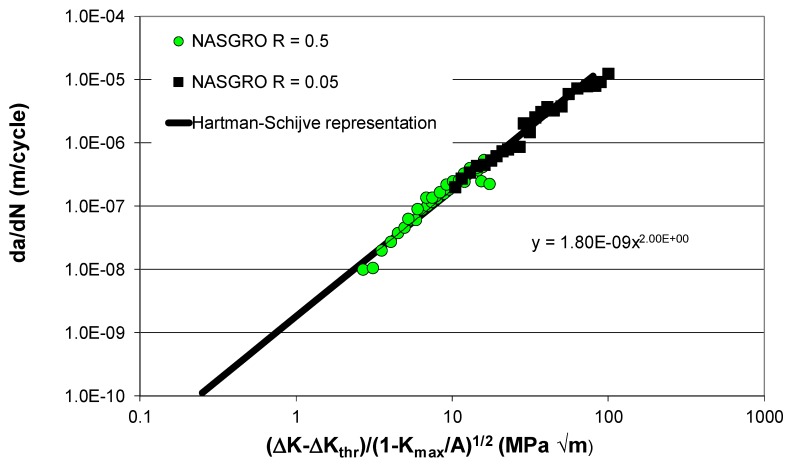
Hartman–Schijve representation of crack growth in AA2024-T3.

Before we could compute the effect of the residual stress on crack growth, we needed the constants *D*, *p* and *A* in Equations (1) and (2) for this thickness material. These constants—*D* = 1.8 × 10^−9^, *p* = 2 and *A* = 45 MPa √m—were obtained from *R* = 0.5 and 0.05 crack growth data that were obtained from the NASGRO database. To do this, we represented the crack growth data by plotting da/dN against Δκ; see Figure 8. The values of ∆*K*_thr_, *D*, *A* and *p* used in this analysis are given in Table 1.

**Figure 9:**
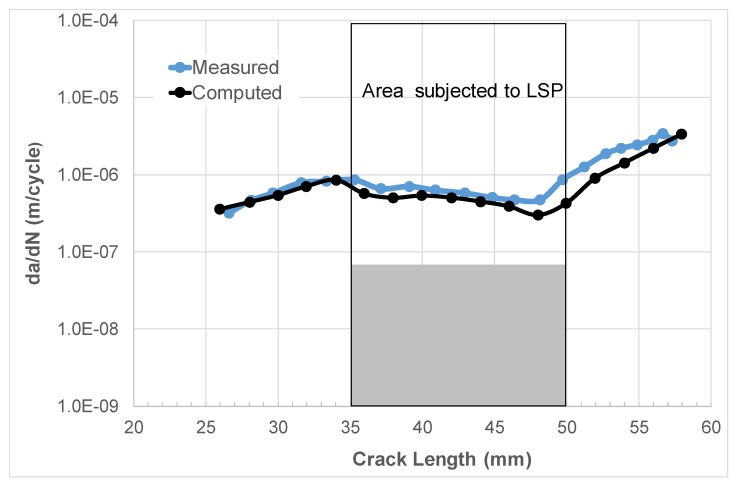
Comparison between the measured [75] and measured da/dN and crack length (*a*) curves.

By using the value of ∆*K*_thr_ (=2.65 MPa √m) associated with the *R* = 0.5 tests and Equation (11), we obtained a value of Δ*K*_thr_ (*R* = 0) equal to approximately 4.65 MPa √m. This value together with the values of *K*_max_, *K*_min_, and *K*_res_ shown in Figure 6 and the values of *D*, *A* and *p* given in Table 1 were then used to compute the relationship between da/dN and the crack length (*a*). A comparison between the computed and measured curves is shown in Figure 9, where we see that, allowing for experimental error, there is a reasonably good agreement between the two curves.

## 5. Effect of Residual Stress on Crack Growth in Wire and Arc Additive Manufactured Ti-6Al-4V

**Figure 10:**
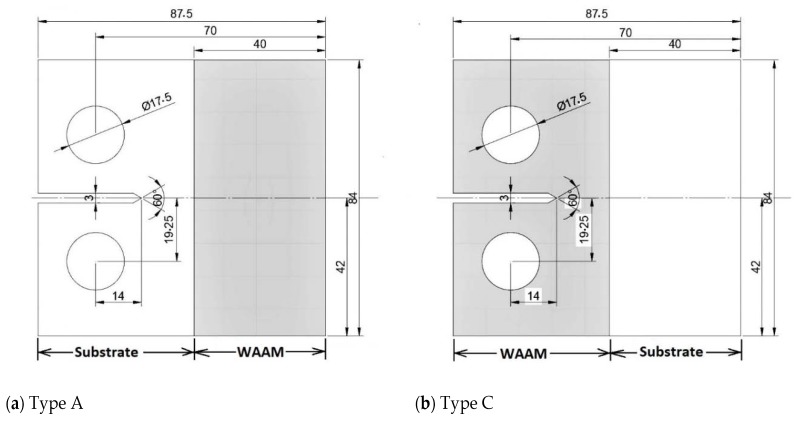
The specimen test geometry used in the Type A, and Type C tests; from [76].

**Figure 11:**
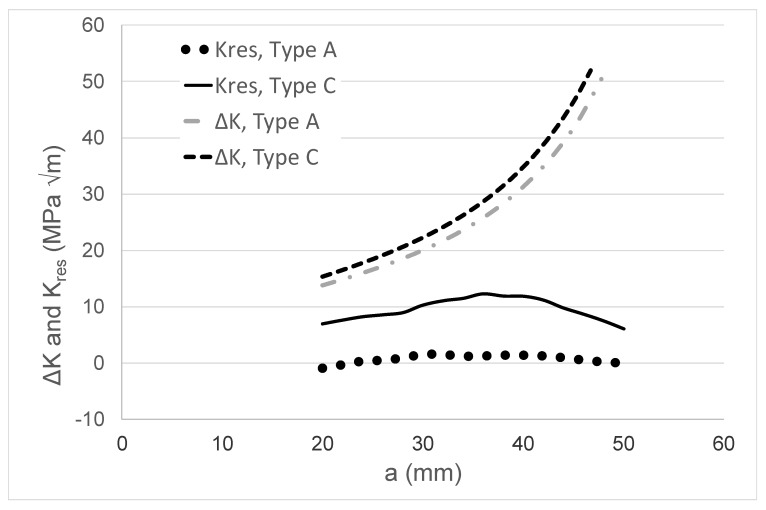
The relationship between the residual stress intensity factors and the crack length (*a*) as determined in [76] for the Type A and Type C specimens.

**Figure 12:**
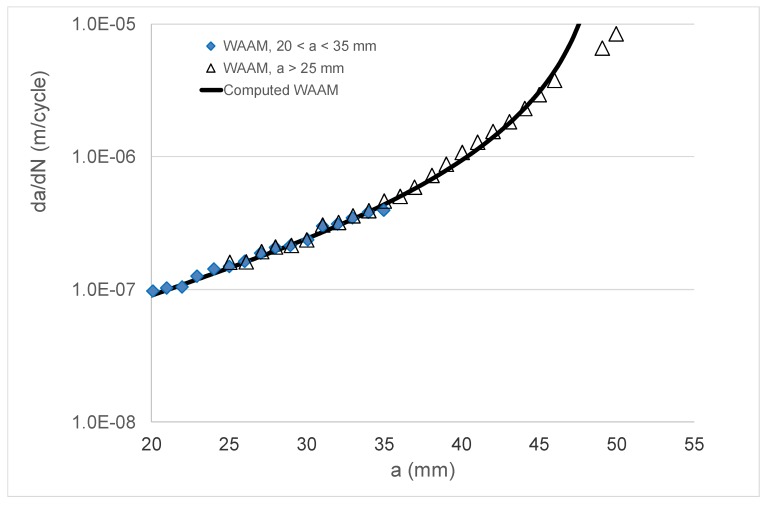
Comparison between the measured and computed growth rate (da/dN) versus crack length (*a*) curves for the wire and arc additive manufactured (WAAM) test specimens.

Having established the ability of the Hartman–Schijve equation to reasonably accurately compute growth in an FSW joint and in an LSP specimen by accounting for the effect of the residual stress field, let us next examine crack growth in tests on a wire and arc additive manufactured (WAAM) Ti-6Al-4V specimen. The paper by Zhang et al. [76] presented *R* = 0.1 da/dN versus Δ*K* data associated with the effect of residual stress on WAAM Ti-6Al-4V CT specimens. One test involved annealed WAAM compact tension specimens. Two additional tests, termed Type A and Type C, were performed on a CT specimen where the WAAM Ti-6Al-4V was deposited on a wrought Ti-6Al-4V substrate; see Figure 10. The tests on specimen Types A and C resulted in a significant residual stress field, as well as a residual stress intensity factor *K*_res_ that varied as a function of the crack length [76]—see Figure 11—that also contains the relationship between ΔK and the crack length. The da/dN versus crack length (a) curves for the WAAM tests are shown in Figure 12.

**Figure 13:**
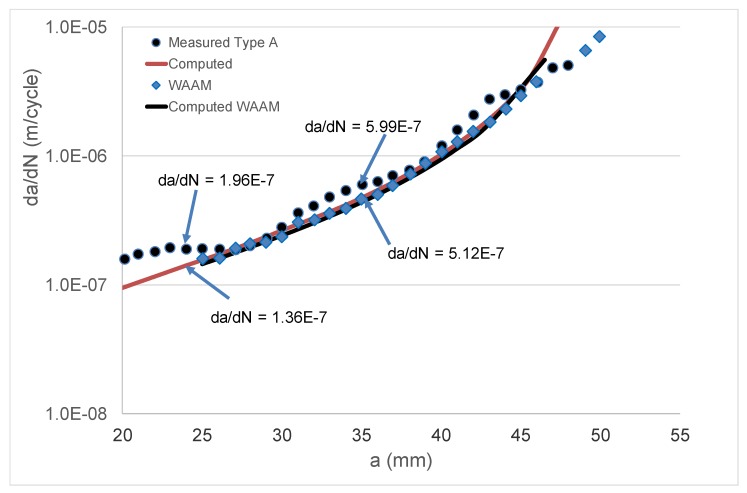
Comparison between the measured and computed growth rate (da/dN) versus crack length (*a*) curves for the Type A specimens.

**Figure 14:**
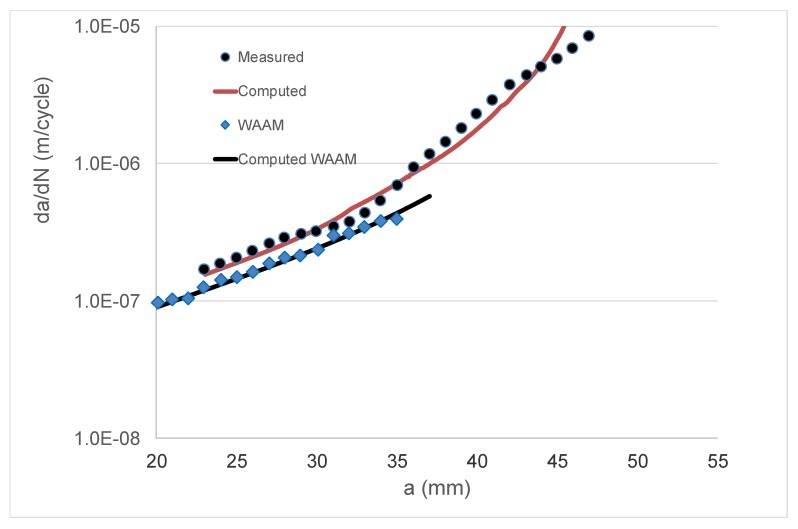
Comparison between the measured and computed growth rate (da/dN) versus crack length (*a*) curves for the Type C specimens.

As previously mentioned, it is now known [3,48,49,50] that crack growth in AM Ti-6Al-4V can be expressed as per Equation (4). As noted in [3], for AM Ti-6Al-4V, the mean value of the parameter *A* in Equation (4) is approximately 62 MPa √m. Indeed, this value is consistent with the crack growth data that were presented by Zhang et al. for this material [77]. This value of *A*, together with a threshold term Δ*K*_thr_ = 0.6 MPa √m, was used to compute the da/dN versus crack length (*a*) curve for the WAAM tests, and a comparison between the measured and computed curves is shown in Figure 12 (a somewhat better fit could have been obtained by slightly increasing the value of the cyclic fracture toughness term (*A*)). Equation (4), together with the value of *A* (=62 MPa √m) given above, was then used to compute the *R* = 0.1 da/dN versus crack length (*a*) curves for the Type A and Type C test specimens. Noting that the natural variability associated with tests on both conventionally manufactured parts [30] and AM Ti-6Al-4V [3,48,49,50] parts can be captured by allowing for small changes in the threshold term (Δ*K*_thr_), as well as the fact that that the crack growth rate in the Type A and Type C tests was somewhat greater than that seen in the WAAM tests, a threshold value of Δ*K*_thr_ = 0.1 MPa √m was used in these analyses. This value was chosen since, as noted in [3,50], it represents an approximate upper bound for the value of Δ*K*_thr_ for AM Ti-6Al-4V parts. In this analysis, the residual stress field was first used to compute the value of *R*_true_, and Equation (11) was then used to determine the value of Δ*K*_true,thr_. The resultant measured and computed da/dN versus crack length (*a*) curves are shown in Figure 13 and Figure 14. Figure 13 and Figure 14 reveal that, allowing for experimental error, the resultant computed and measured da/dN versus crack length (*a*) curves are in reasonably good agreement. This represents an interesting finding in that it suggests that even in the presence of residual stresses, crack growth in AM Ti-6Al-4V can be computed by using the crack growth equation that was determined for conventionally manufactured Ti-6Al-4V, i.e., Equation (4), provided that allowance is made for the effect of the residual stresses.

## 6. Residual Stresses: AM Replacement Parts and Cold Spray

Though this paper initially concentrated on the growth of long, artificially induced cracks, it should be recalled that the airworthiness certification of AM replacement parts requires a durability analysis [4] and that the EIDS that is required for the associated durability analysis is generally sub mm [4]. Indeed, as first shown by Lincoln and Melliere [78] as part of the USAF F-15 program and subsequently highlighted in [3,6,79,80,81,82], a durability analysis necessitates the use of the associated small crack da/dN versus Δ*K* curve (a similar statement is contained in Appendix A3 of the ASTM fatigue test standard E647-13a [83]). In this context, as noted in [6], the experimental data revealed that the R ratio effect associated with the growth of small, naturally occurring cracks is often quite small. This in turn suggests that the effect of the residual stresses on the growth of small, naturally occurring cracks in an AM part may also be small. This hypothesis is consistent with the findings that were reported in [84] for the growth of small cracks in annealed cast AM60 magnesium alloy specimens, where it was stated that the “Maximum stress did not appear to affect the crack propagation rate of small cracks in the stress and crack size ranges considered,” and in [50], where it was shown that the growth of small cracks in a cast rail steel was essentially independent of the R ratio. Interestingly, the authors of [50] also revealed that the da/dN versus Δ*K* curves associated with for the growth of small cracks in LENS Ti-6Al-4V essentially coincided with that seen in this particular cast steel. Unfortunately, there are currently insufficient data to assess the R ratio independence of small crack growing in an AM part.

**Figure 15:**
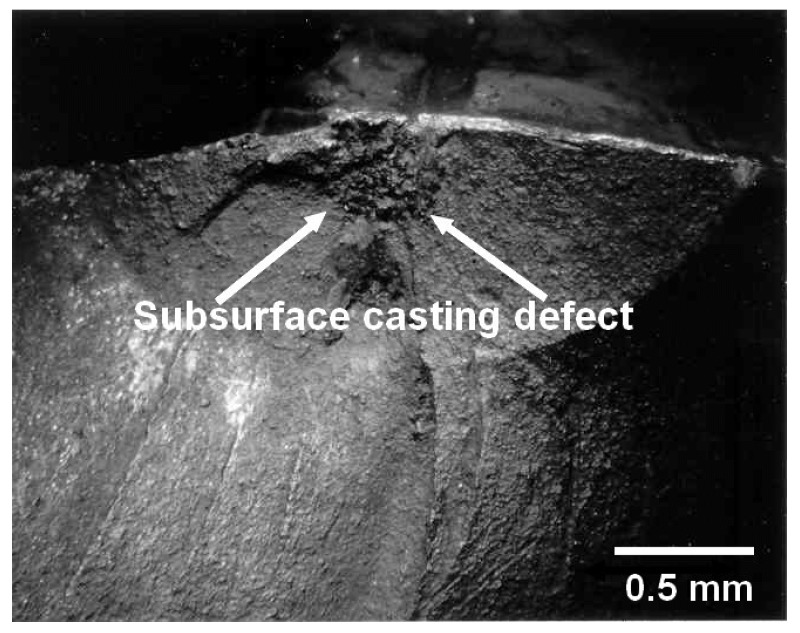
Typical subsurface defect at the inner pedestal leg of a side frame.

The durability analysis of AM replacement parts and additive metal repairs needs to consider both surface breaking and near surface material discontinuities, e.g., a lack of fusion. Whilst it is now generally acknowledged that for laboratory tests on flat AM coupons, surface breaking material discontinuities are the most critical [3,6,85,86,87,88], this is not necessarily true for parts with complex geometries. This conclusion is aptly illustrated by the cast steel rail side frame failures reported in [89], where failure was often initiated as a result of the interaction between (near) subsurface material discontinuities and the local geometric stress concentrator; see Figure 15. Consequently, the question of the effect of residual stresses on near surface material discontinuities needs to be resolved. In this context, it should be noted that the seminal paper by Schijve [90] established that the reduction in the growth rate of interior (sub-surface) cracks in conventionally manufactured material was due to the environment, with interior cracks growing in a “vacuum-like” environment. It is now known [91,92] that cracks growing in a near vacuum-like environment see both an enhanced fatigue threshold and a greatly reduced R ratio effect. These observations suggest that the effect of the residual stresses on the growth of small, naturally occurring sub surface material discontinuities in an AM part should be less than for surface breaking material discontinuities. Unfortunately, there are insufficient data to investigate this hypothesis.

**Figure 16:**
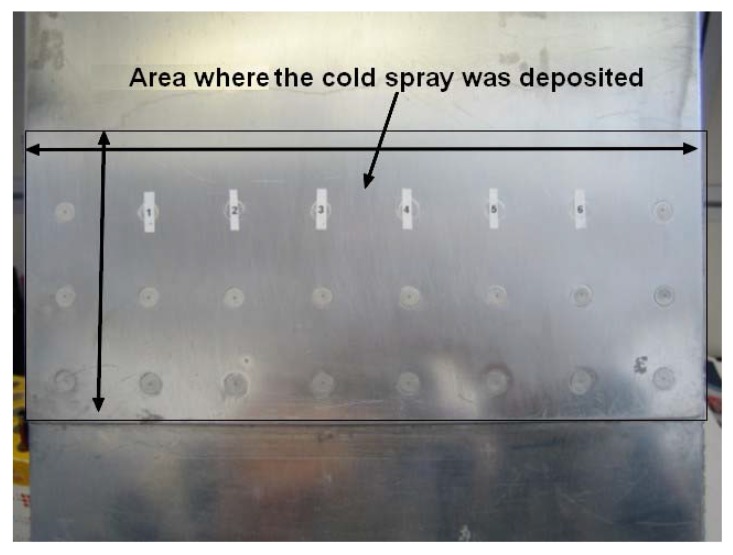
Plan view a fuselage lap joint specimen panel and fastener numbers showing the area over which the cold spray was deposited; from [93].

**Figure 17:**
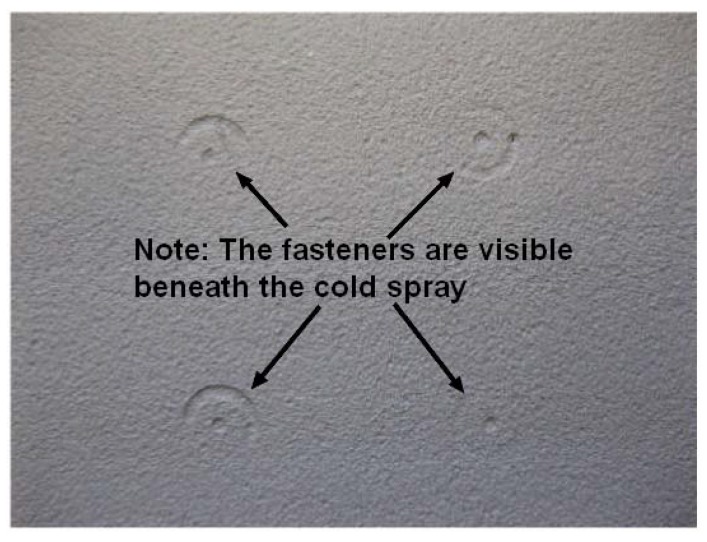
A close up view of a cold spray repair over fasteners; from [93].

**Figure 18:**
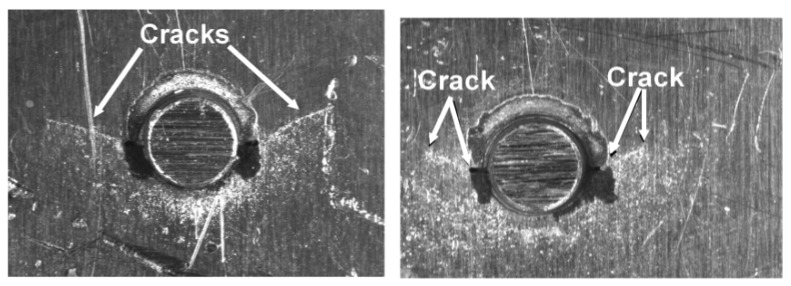
View of the underside (on which the cold spray was not deposited) of the 2024-T3 lap joint skin, where there was no cracking in the cold spray; from [93].

The situation is somewhat different for cold spray repairs, also known as SPD repairs, to aluminum alloy airframes. For cold spray repairs deposited by using 7075 and 6061 aluminum alloy powders, it is generally found that, provided that the repair is not heat treated, the compressive residual stresses induced in the cold spray by the deposition process inhibits the development of surface breaking cracks [46,47,93]. Indeed, as shown in [46,93] cold spray repairs are often very resistant to cracking in the underlying airframe. An example of this is shown in Figure 16, Figure 17 and Figure 18, which presents the results of a study [93] into cold spray repairs to multi-site damage in fuselage lap joints representative of that in Boeing 737 aircraft. In these tests, each fastener hole contained initial cracks that did not extend beyond the head of the (5/32 inch diameter) countersink rivet and hence were not visible; see [94] for more details. The geometry of the baseline test specimen, i.e., a specimen without cold spray repair, is shown in Figure 16; from [93]. An important feature of this test program is that the design of the baseline specimen was such that it reproduced the crack growth history seen in the US Boeing 737 fleet [94]. Specimens with and without cold spray repairs were tested. The cold spray repair covered the three rows of fasteners in the top skin; see Figure 16 and Figure 17. In this test program, it was found that the residual compressive stresses in the cold spray meant that it could tolerate cracks of up to 6 mm in length in the underlying 2024-T3 skin without cracking; see Figure 18.

As a result, it appears that, provided that the repair is not heat treated, cracking in cold spray repairs tends to develop either in the airframe (i.e., the underlying structure) or at the intersection between the cold spray repair and the airframe (substrate); see [46,62,95]. As such, the question of the effect of residual stresses on the growth of subsurface material discontinuities is of particular relevance for cold spray repairs.

**Figure 19:**
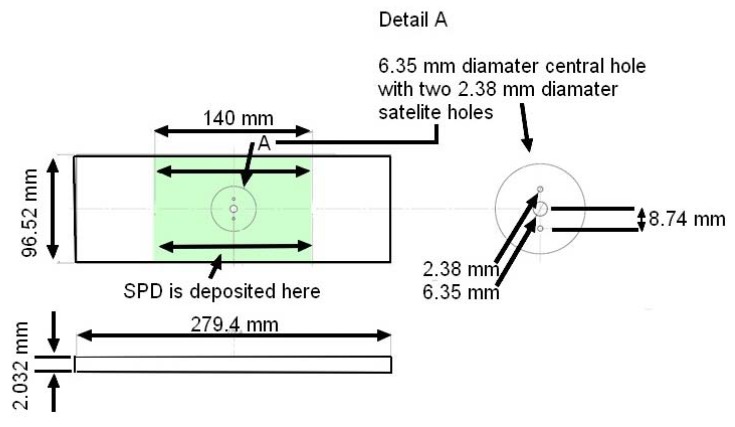
Schematic diagram of the supersonic particle deposition (SPD)-repaired dome nut fastener hole (DNH) coupons; from [46].

**Figure 20:**
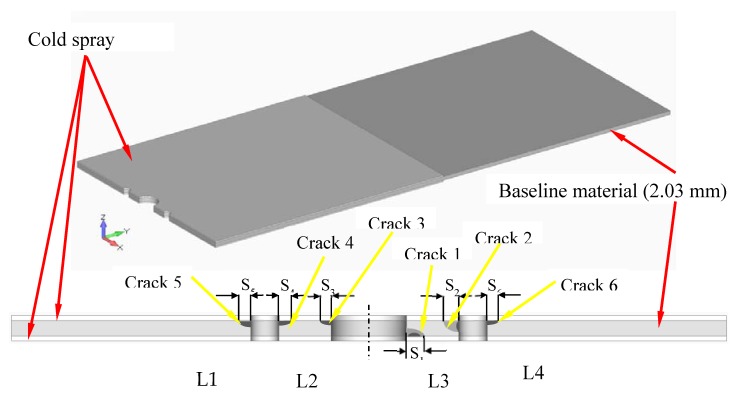
Schematic of ½ of a dome nut fastener hole specimen (DNHS) coupon with a 0.453 mm-thick cold spray repair on either side of the test specimen; from [46].

In this context, the specimens test program reported in [46] addressed the use of cold spray to repair when using 7075 powder on specimens containing a dome nut fastener hole that were cut from sections of an approximately 2.0 mm-thick AA7075-T6 P3C Orion wing skin; see Figure 19 and Figure 20. These test specimens contained intergranular cracking at the fastener hole; see [46] for more details. This study revealed that cracks could nucleate either at the bore of the holes near the intersection between the cold spray and the 7075-T6, or away from the fastener holes at the intersection between the cold spray and the 7075-T6 substructure. This is aptly illustrated in Figure 20, which illustrates the initiation locations for six cracks, which are labelled Crack 1 to Crack 6, in test specimen dome nut fastener hole specimen (DNHS)-1-12. The size of the initial material discontinuities (cracks) seen in these tests lay in the range (approximately) 0.02 to 0.045 mm; see [46] for more details. Specimen DNHS-1-12 was subjected to a series repeated load blocks. Each load block consisted of 3000 cycles at *R* = 0.1, and 15,000 cycles at *R* = 0.8, with the maximum remote stress (σmax) of 150 MPa being held constant throughout the test.

While [46] compared the computed and measured crack growth histories associated with the fastest growing cracks in seven different specimen tests, it did not present the crack growth histories associated with each of the six cracks seen in DNHS-1-12. The analysis methodology that was used in [46] used the approach presented in [96,97], which was based on the three dimensional multi-crack finite element alternating technique [98] and, as such, did not require the cracks to be explicitly modelled to determine the stress intensity factor versus crack geometry solutions for each of the specimen tests. The present paper also used this methodology to determine the stress intensity factor histories associated with each of the six cracks seen in DNHS-1-12.

As in [31,46], the crack growth analysis used the stress intensity factor solutions as input to the Hartman–Schijve crack growth equation for AA7075-T6 [31,46]:

da/dN = 1.86 × 10^−9^ [(Δ*K* − Δ*K*_thr_)^2^/(1 − *K*_max_/111) (12)

**Figure 21:**
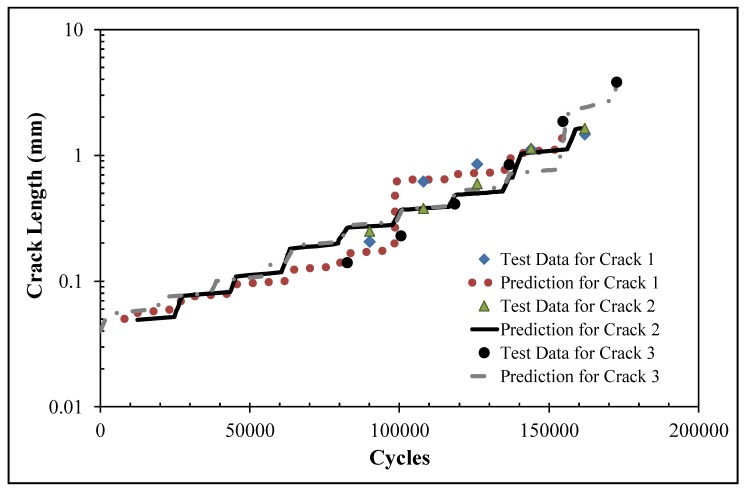
Measured and computed crack length histories for cracks 1–3 in specimen DNHS-1-12.

**Figure 22:**
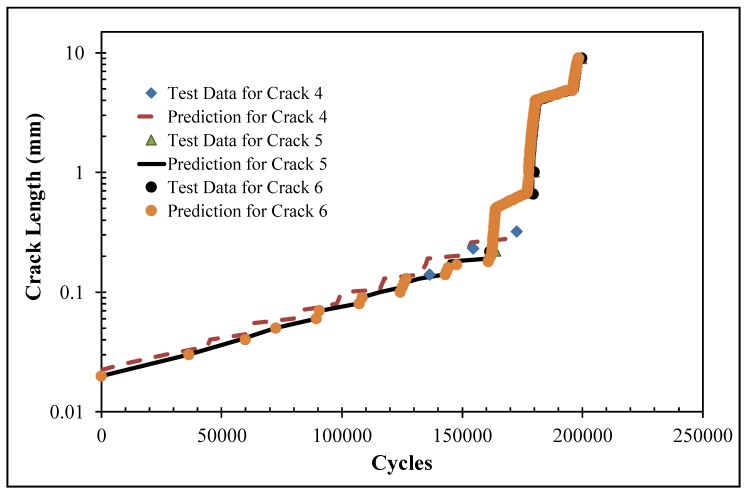
Measured and computed crack length histories for cracks 4–6 in specimen DNHS-1-12.

As per [50], the value of the threshold term Δ*K*_thr_ that was used in the analysis of each of these six (small) cracks was taken to be 0.2 MPa √m. The resultant measured and computed crack length histories for cracks 1–3 and cracks 4–6 are shown in Figure 21 and Figure 22, respectively, where it can be seen that, in each case, the agreement was quite good. At this point, it should be stressed that this is the first study to show how to compute the growth of small, naturally occurring collocated cracks in an AM repair and that is a requirement that is enunciated in Structures Bulletin EZ-19-01 [4].

As such, it appears that as in [46], which found that the crack growth histories associated with the fastest growing (small) cracks in seven different cold spray repair specimens could be captured without allowing for the effect of the residual stresses that are induced both in the cold spray and the substrate as a result of the deposition process, the growth of these six different co-located small cracks was also captured reasonably well when the effect of the residual stresses was ignored (at this stage, it should be noted that the paper by White et al. [62], which examined cold spray repairs to corroded fastener holes, also reported that failure was due to cracks growing either at the interface between the substrate and the cold spray or in the substrate). Consequently, the study presented in [62], when taken in conjunction with the results presented in [46], and the results presented above appear to suggest that the residual stresses induced in the 7075-T6 substrate by the cold spray deposition process do not appear to have a particularly detrimental influence on the growth of small, naturally occurring cracks that nucleate either in the substrate or at the interface between the substrate and the cold spray.

Unfortunately, despite the need to predict the growth of cracks in cold spray repairs that nucleate from small, naturally occurring material discontinuities at the cold spray-substrate interface other than the analyses presented in [46] and in the present paper, there are, to the best of the author’s knowledge, no papers that attempt to analyze this phenomenon. It is thus suggested that further work is required to address the question of the effect of residual stresses on the growth of small, naturally occurring material discontinuities in AM parts and on the growth of small, naturally occurring material discontinuities at the intersection between a cold spray repair and the airframe.

On a related topic, it should be noted that the experimental data presented in [46,99] also suggest that the buckling load of panels containing either skin corrosion or stress corrosion cracking in the risers that are repaired by using cold spray is also relatively unaffected by the induced residual stress.

## 7. AM Illustration of the Use of EIDS to Compute the Growth of Multiple Cracks from A Rough Surface

The Airbus study [100] into the effect of surface roughness on the fatigue performance of AM Ti-6Al-4V noted that surface roughness often results in the initiation of cracks at multiple sites. USAF Structures Bulletin EZ-19-01 [4] also highlights the need to develop a fracture mechanics-based methodology for the analysis of the growth of multiple surface breaking cracks from a rough surface. In the present investigation, two AA7050-T7451 dog-bone shaped specimens were tested to investigate crack growth from an array surface material discontinuities. The specimens were 380 mm long, 60 mm wide, and 11 mm thick. The specimens were first polished to remove the oxidation layers on the surface and surface scratches and then cleaned by using methyl alcohol.

**Figure 23:**
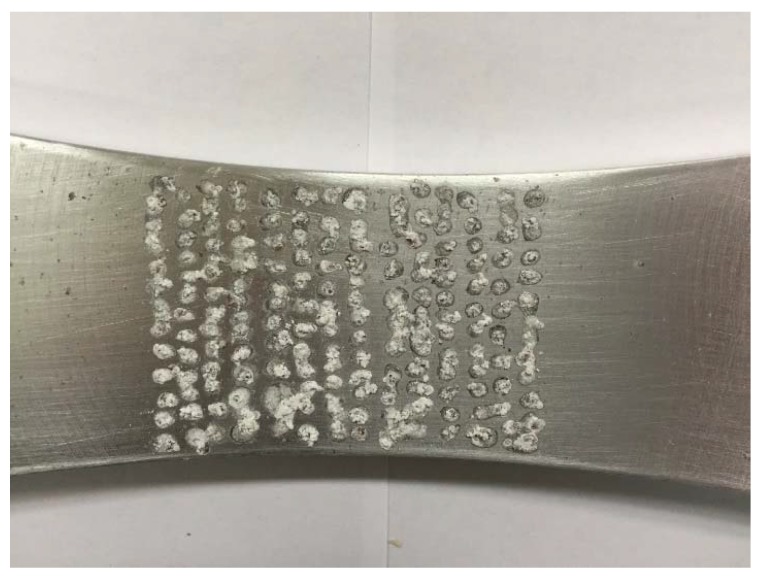
View of a typical surface.

In an attempt to mimic a rough surface with multiple collocated potential initiation sites, it was decided to create an array of surface pits. To produce an array of pits, with depths and diameters comparable with the EIDS requirement, given in USAF Structures Bulletin EZ-19-01 [4], of a minimum size of 0.254 mm (0.01 inch) a total of 12 by 14 drops of a 3.54% NaCl solution were introduced on both the upper and lower surfaces of the specimens. The spacing between each location was approximately 3.5 mm. This meant that there was a total of 336 possible initiation sites. The specimens were then kept in a sealed container for six days. A bottle of supersaturated K2SO4 solution was placed in the container to maintain the moisture and hence prevent the evaporation of the “drops.” After six days of “corrosion,” the specimens were dried at 50 °C in an oven for an hour. An example of a resultant “rough” surface created by this process is shown in Figure 23.

**Figure 24:**
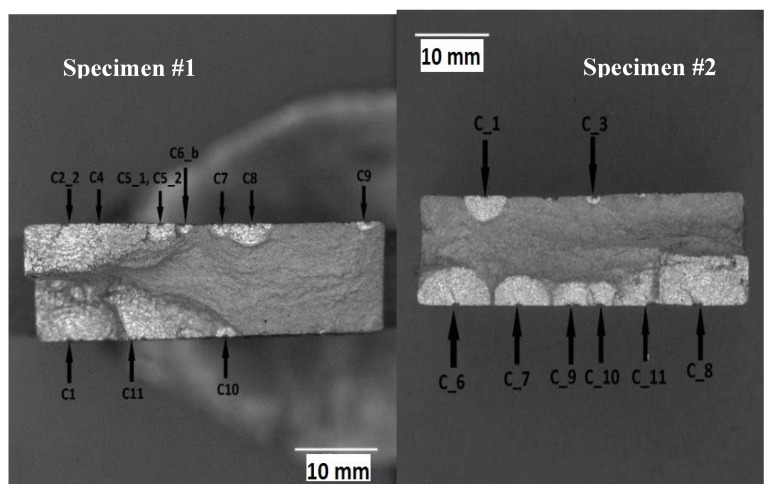
Fracture surface and schematic locations of cracks in Specimen #1 (left) and Specimen #2 (right).

The specimens were subsequently fatigue tested under constant amplitude loading with a marker band inserted at the end of each block. Each load block consisted of 15,000 cycles at R = 0.1 and 300 cycles at R = 0.8. The maximum load associated with the R = 0.1 and 0.8 cycles was kept constant at 139.92 kN. The tests were performed at 5Hz in a (laboratory) air environment. The 300 cycles at R = 0.8 were introduced to aid in quantitative fractography. Specimen #1 failed after 505,000 cycles of load, i.e., 34 load blocks. Specimen #2 failed after 489,690 cycles, i.e., 32 load blocks; see Figure 24.

**Figure 25:**
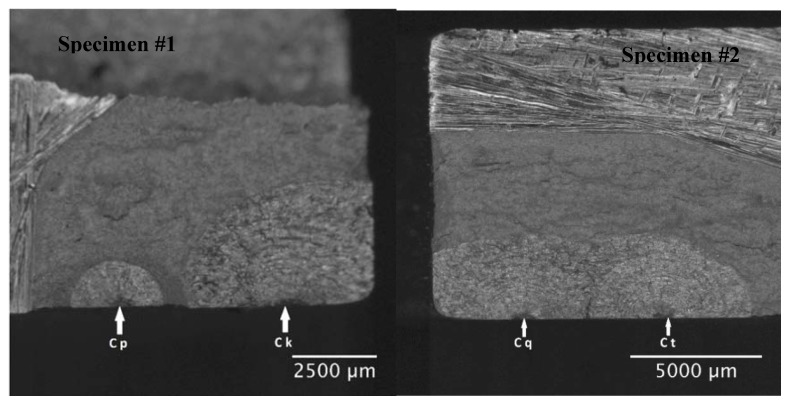
The two cracks on the manually opened surfaces of Specimen #1 (left) and Specimen #2 (right).

**Figure 26:**
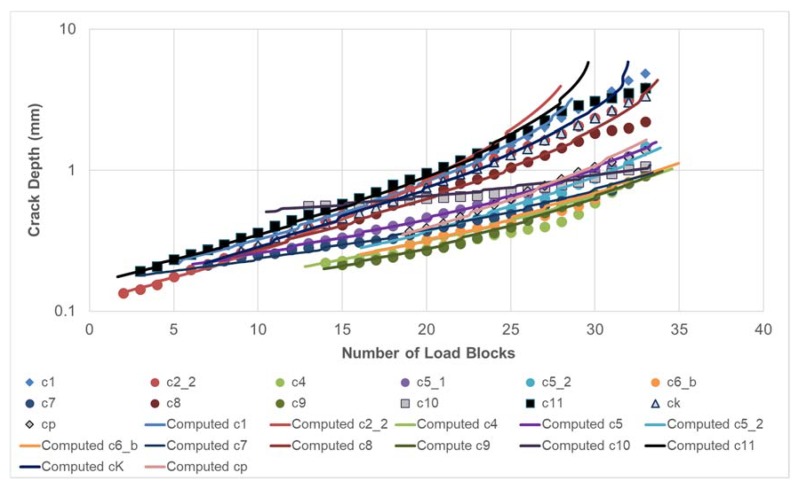
Comparison between the measured and computed crack growth histories for Specimen 1.

**Figure 27:**
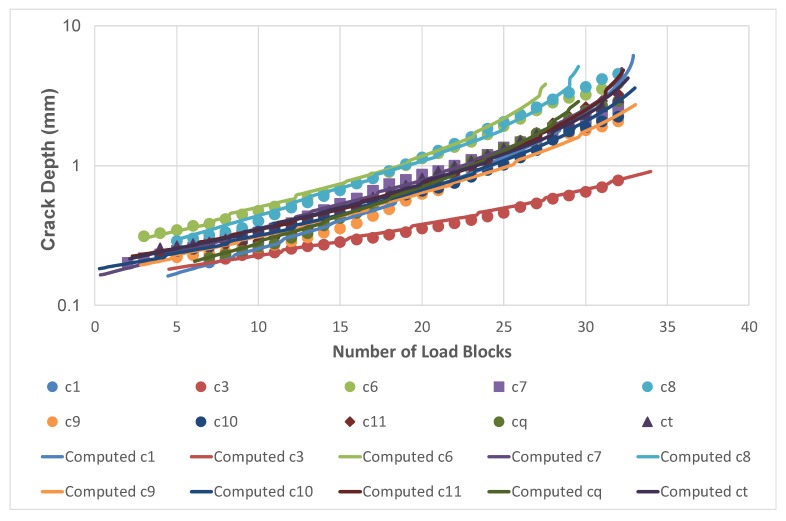
Comparison between the measured and computed crack growth histories for Specimen 2.

Quantitative fractography analysis was subsequently used to determine the crack growth histories of each of the eleven cracks seen on the fracture surface of Specimen 1 and the eight cracks seen on the fracture surface of Specimen 2. These cracks were labelled as shown in Figure 24. It was subsequently found that there were four cracks that were not fully opened after fatigue testing. These cracks, which had grown to a surface length of approximately 3 mm, are shown in Figure 25. The crack growth histories associated with these twenty three surface breaking cracks are shown in Figure 26 and Figure 27. The size of the initiating material discontinuities (pits) was also measured, and details of the twenty three surface pits (including initial surface length, depth and location from the edges of the specimen) are given in Table 2. The mean initial depth (a_i_) was 0.195 mm, and the standard deviation was 0.052 mm. The maximum depth was approximately 0.33 mm. The mean surface half length (c_i_), at the fracture plane, was 0.315 mm, and the standard deviation was 0.115 mm. The maximum value of c_i_ was approximately 0.437 mm. The mean aspect ratio (=c_i_/a_i_) was approximately 1.64 with a standard deviation of 0.45. As such, the depths and surface lengths were comparable with the minimum EIDS required in [4] of 0.254 mm (0.01 inch).

The crack growth history for each of these twenty cracks was then computed by using the Equation (1). The values of *D*, *p*, and *A* that were used in this analysis were taken from [9] and were: *D* = 7 × 10^−10^, *p* = 2, and *A* = 47 MPa √m. The values of ∆K*_thr_* that were used in the analyses are given in Table 3.

The resultant measured and computed fatigue crack depth histories, with the initial crack sizes assumed to be as per Table 2, for each of the twenty three cracks are shown in Figure 26 and Figure 27. Here, we see that the variability in the crack growth histories was captured reasonably well by using an EIDS that was comparable with the measured initial defect sizes. The mean value of Δ*K_thr_* in these analyses was 0.8 MPa√m, with a standard deviation of 0.24 MPa√m. The maximum and minimum values of Δ*K_thr_* used in the analysis were 1.6 and 0.35 MPa√m, respectively. The value of Δ*K_thr_* = 0.35 MPa√m was associated with the fastest growing crack.

## 8. Conclusions

USAF Structures Bulletin EZ-19-01 [4] highlights the need to develop a fracture mechanics-based methodology for certifying both AM parts and AM repairs. Specific mention is made of the need for the analysis tools to be able to allow for manufacturing induced residual stresses and for multiple collocated cracks. This paper presented examples of how to perform such an analysis. To this end, the present paper first examined a range of examples that illustrate the potential of the Hartman–Schijve crack growth equation to reasonably-well account for the effect of residual stresses on the growth of long cracks in tests on laser shock peened and friction stir welded specimens. These studies illustrate that when allowance is made for the effect that the residual stress intensity factor K_res_ has upon the true range of the stress intensity factor (∆Ktrue) and on the true value of the maximum stress intensity factor (Ktrue,max) then crack growth can be reasonably accurately computed. Here, the approach was then shown to hold for AM Ti-6Al-4V specimens.

The present paper also illustrated how this approach can also be used to perform the fracture mechanics-based durability analyses required in Structures Bulletin EZ-190-01 for a cold spray repair where there are multiple cracks and where the initiating defect sizes are comparable with the EIDS sizes presented in Structures Bulletin EZ-19-01. It was also shown how the Hartman–Schijve crack growth equation can be used to compute the crack growth histories associated with multiple cracks that nucleate from a rough surface where the initiating defect sizes are comparable with both EIDS sizes presented in Structures Bulletin EZ-19-01.

## Figures and Tables

**Figure 2:**
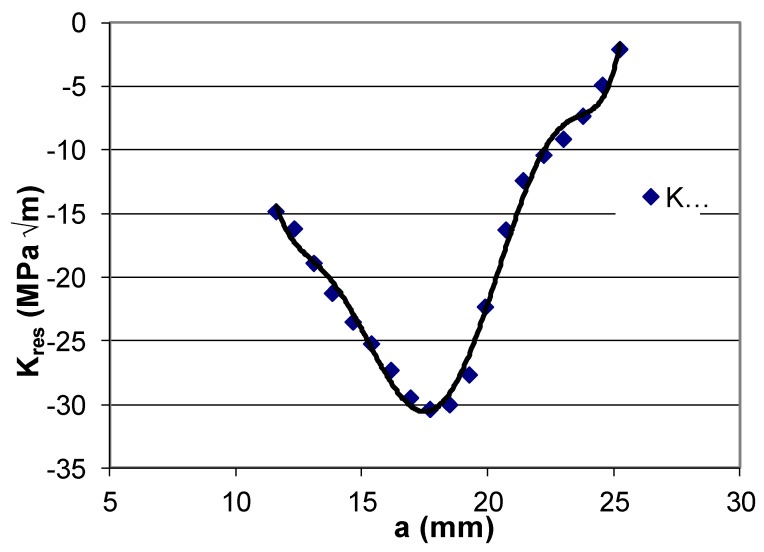
The dependency of residual stress intensity factor (*K*_res_) versus crack length (*a*); from [72].

**Table 1:** The values of ∆*K*_thr_ used in Figure 8.

	∆*K*_thr_ (MPa √m)	*D*	*A* (MPa √m)
*R* = 0.5	2.65	1.8 × 10^−9^	45
*R* = 0.05	4.0	1.8 × 10^−9^	45

**Table 2:** Details of the twenty three surface material defects in Specimens 1 and 2.

Specimen	Crack	c_i_ (mm)	a_i_ (mm)	Aspect Ratio (=c_i_/a_i_)	Distance to Edge (mm)
1	c1	0.271	0.124	2.188	4.2
	c2_2	0.129	0.11	1.173	4.9
	c4	0.272	0.194	1.402	8.8
	c5_1	0.25	0.19	1.316	16.5
	c5_2	0.25	0.2	1.250	16.5
	c6_b	0.31	0.24	1.292	19
	c7	0.252	0.169	1.488	20.5
	c8	0.35	0.22	1.591	16.3
	c9	0.185	0.176	1.051	2.4
	c10	0.336	0.232	1.448	19.5
	c11	0.25	0.122	2.050	12.2
	ck	0.407	0.2	2.035	2.5
	cp	0.39	0.328	1.189	7.5
2	c1	0.26	0.15	1.733	8.9
	c3	0.321	0.169	1.896	20.5
	c6	0.437	0.285	1.533	5.2
	c7	0.414	0.15	2.760	13.8
	c8	0.7	0.269	2.602	6.7
	c9	0.31	0.18	1.722	20.6
	c10	0.23	0.174	1.322	20.2
	c11	0.271	0.212	1.278	13
	cq	0.29	0.191	1.518	3.5
	ct	0.369	0.199	1.854	8.7

**Table 3:** Values of the threshold term (Δ*K_thr_*) that were used in the analysis.

Specimen	Crack	Δ*K_thr_* (MPa√m)
1	c1	0.7
	c2_2	0.35
	c4	0.75
	c5_1	0.8
	c5_2	0.6
	c6_b	0.75
	c7	0.85
	c8	0.72
	c9	0.65
	c10	1.6
	c11	0.61
	ck	0.83
	cp	0.63
2	c1	0.6
	c3	0.95
	c6	1
	c7	0.9
	c8	1.2
	c9	0.8
	c10	0.68
	c11	0.75
	cq	0.66
	ct	0.92

## Appendix A

The crack growth equation given by McEvily and Groeger [69] can be written in the form:

da/dN = D [(Δ*K* − Δ*K*_thr_) (1 + Δ*K*/(*K*_c_ − *K*_max_))^1/2^]^2^ (A1)

where *K*_c_ is the cyclic fracture toughness. The role of the term (1 + Δ*K*/(*K*_c_ − *K*_max_)) in Equation (A1) is to increase the crack growth rate when *K*_max_ approaches *K*_c_. McEvily and Groeger [69] revealed that Equation (A1) led to the following expression for Δ*K*_thr_:

Δ*K*_thr_ = Δ*K*_thr_(*R* = 0) [(1 + *R*)/(1 + *R*)]^1/2^ (A2)

where the term Δ*K*_thr_(R = 0) denotes the value of Δ*K*_thr_ at *R* = 0.

In the tests studied in the present paper, the R ratio associated with the applied load was 0.1. These tests enabled the value of Δ*K*_thr_ at *R* = 0.1, which in this section, we denote as Δ*K*_thr_(*R* = 0.1), to be determined. This value can be used together with Equation (A2) to estimate the value of Δ*K*_thr_(R = 0):

Δ*K*_thr_(*R* = 0) = 0.9045 Δ*K*_thr_(*R* = 0.1)
(A3)

The value of the fatigue threshold at the various effective R ratio values can now be estimated by using Equation (A2).
